# Microglia Heterogeneity in Alzheimer’s Disease: Insights From Single-Cell Technologies

**DOI:** 10.3389/fnsyn.2021.773590

**Published:** 2021-12-23

**Authors:** Hansen Wang

**Affiliations:** Leslie Dan Faculty of Pharmacy, University of Toronto, Toronto, ON, Canada

**Keywords:** microglia, microglia heterogeneity, Alzheimer’s disease, single-cell sequencing, single-cell RNA sequencing, single-nucleus RNA sequencing, neuroinflammation, neurodegenerative diseases

## Abstract

Microglia are resident immune cells in the central nervous system and play critical roles in brain immunity, development, and homeostasis. The pathology of Alzheimer’s disease (AD) triggers activation of microglia. Microglia express many AD risk genes, suggesting that their response to AD pathology can affect disease progression. Microglia have long been considered a homogenous cell population. The diversity of microglia has gained great interest in recent years due to the emergence of novel single-cell technologies, such as single-cell/nucleus RNA sequencing and single-cell mass cytometry by time-of-flight. This review summarizes the current knowledge about the diversity/heterogeneity of microglia and distinct microglia states in the brain of both AD mouse models and patients, as revealed by single-cell technologies. It also discusses the future developments for application of single-cell technologies and the integration of these technologies with functional studies to further dissect microglia biology in AD. Defining the functional correlates of distinct microglia states will shed new light on the pathological roles of microglia and might uncover new relevant therapeutic targets for AD.

## Introduction

Microglia, the predominant resident immune cells within the central nervous system (CNS), are capable of performing various functions in the brain under both homeostatic and disease conditions (Hansen et al., 2018; Tejera and Heneka, 2019; Bartels et al., 2020; Leng and Edison, 2021). Alzheimer’s disease (AD) is an age-related neurodegenerative disease with progressive memory decline and cognitive dysfunction, which is pathologically characterized by extracellular deposition of β-amyloid (Aβ) and intracellular neurofibrillary tangles (NFT) of hyperphosphorylated tau, accompanied by neuroinflammation, neuronal, and synapse loss (Long and Holtzman, 2019; van der Kant et al., 2020; Knopman et al., 2021). Microglia is a critical cellular player in neuroinflammation. Changes in microglial morphology and density as well as increased expression of microglia activation markers have been well documented in AD (Hansen et al., 2018; Prinz et al., 2019; Tejera and Heneka, 2019; Bartels et al., 2020; Streit et al., 2021). Activated or functionally changed microglia have been observed in pathologically relevant brain regions of both AD mouse models and patients (Hansen et al., 2018; Tejera and Heneka, 2019; Bartels et al., 2020; Streit et al., 2021). In addition, genetic evidence has directly linked microglial function to AD. Many of AD risk genes, such as triggering receptor expressed in myeloid cells 2 (*TREM*2), complement receptor 1 (*CR1*), cluster of differentiation 33 (*CD33*) and inositol polyphosphate-5-phosphatase (*INPP5D*), are preferentially expressed in microglia (Shi and Holtzman, 2018; Verheijen and Sleegers, 2018; Tejera and Heneka, 2019; Hashemiaghdam and Mroczek, 2020; Leng and Edison, 2021; Streit et al., 2021). Functional studies of these AD risk genes have been instrumental in establishing roles of microglia in AD pathogenesis and progression (Shi and Holtzman, 2018; Ulland and Colonna, 2018; Sierksma et al., 2020; Bhattacherjee et al., 2021; Chen and Colonna, 2021; Lee et al., 2021). However, it is still unclear whether microglial function in AD is beneficial but insufficient, or whether these cells function differently at early and late disease stages (Deczkowska et al., 2018; Hashemiaghdam and Mroczek, 2020; Lewcock et al., 2020; Chen and Colonna, 2021; Leng and Edison, 2021; Streit et al., 2021). Much remains to be learned about the phenotypes and functions of microglia, and the molecular changes underlying the responses of microglia in AD brain.

The characterization of microglial phenotypes under disease conditions has been a research focus for years. Numerous studies that characterized the features of microglia, such as their density and morphology, have indicated that microglia are heterogeneous and dynamic. Genome-wide transcriptional profiling of microglia with bulk RNA-sequencing (RNA-seq) has revealed temporal, brain regional and gender-dependent heterogeneity of these cells in neurodegenerative diseases (Hansen et al., 2018; Tejera and Heneka, 2019; Hashemiaghdam and Mroczek, 2020; Leng and Edison, 2021). However, the expression profiling of microglia in bulk cannot reflect the responses of individual cells or reveal microglia subclusters, highlighting that the heterogeneity of microglia needs to be investigated at single-cell resolution (Dorman and Molofsky, 2019; Gerrits et al., 2020; Hashemiaghdam and Mroczek, 2020; Chen and Colonna, 2021; Provenzano et al., 2021). Notably, traditional single-cell analyses of microglia, using techniques, such as cell flow cytometry, *in situ* hybridization, or immunohistochemistry, that are limited to sorting cell populations according to a small set of canonical cell-surface markers, might obscure the presence of additional microglia subtypes and overlook the dynamic diversity of these cells in the brain, greatly hindering the ability to build a comprehensive overview of microglia heterogeneity and complexity (Colonna and Brioschi, 2020; Masuda et al., 2020; Chen and Colonna, 2021; Provenzano et al., 2021; Young et al., 2021).

The advent of single-cell RNA-seq (scRNA-seq) has enabled the profiling of single cells with high-throughput datasets and the defining of microglia clusters based on their transcriptional signatures. In parallel, single-nucleus RNA-seq (snRNA-seq) has allowed transcriptomic analysis of single cells from postmortem human tissues (Keren-Shaul et al., 2017; Chew and Petretto, 2019; Hammond et al., 2019; Li et al., 2019; Gerrits et al., 2020; Masuda et al., 2020; Ndoja et al., 2020; Zhou et al., 2020; Chen and Colonna, 2021; Yang et al., 2021). Additionally, single-cell mass spectrometry [cytometry by time-of-flight (CyTOF)] currently allows the analysis of more than 50 different surface markers at single-cell level (Colonna and Brioschi, 2020; Masuda et al., 2020; Chen and Colonna, 2021; Provenzano et al., 2021). These new single-cell technologies have greatly enriched our knowledge of microglial responses in AD and other neurodegenerative diseases, leading to the identification of special microglia populations associated with neurodegeneration (Dorman and Molofsky, 2019; Olah et al., 2020; Chen and Colonna, 2021; Provenzano et al., 2021; Wang et al., 2021; Young et al., 2021).

This review will provide a description of recent studies that explore microglia heterogeneity using advanced single-cell technologies in the brain of both AD mouse models (Table 1) and patients (Table 2). These studies are helping to identify novel markers, pathways, and regulatory factors that are critical for the function of microglia and might eventually become therapeutic targets for AD.

**TABLE 1:** Single-cell analysis of microglia in Alzheimer’s disease mouse models.

References	Mouse model	Method	Brain region	Microglia state/cluster (gene signature)
Keren-Shaul et al., 2017	5xFAD	scRNA-seq	Cortex and cerebellum	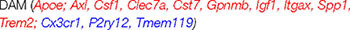
Mathys et al., 2017	CK-p25	scRNA-seq	Hippocampus	DAM like subset; reactive microglia subsets with IFN I and II response genes
Mrdjen et al., 2018	*APP/PS1*	CyTOF	Whole brain	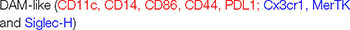
Sala Frigerio et al., 2019	*App* *^NL–G–F^*	scRNA-seq	Cortex and hippocampus	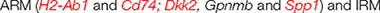
Sierksma et al., 2020	*APP^swe^*/*PS-1*^L166P^** and Thy-TAU22	scRNA-seq	Hippocampus	ARM enriched for AD risk genes
Zhou et al., 2020	5xFAD	snRNA-seq	Cortex and hippocampus	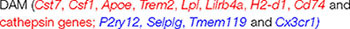
Lee et al., 2021	*TauPS2APP*	scRNA-seq	Hippocampus	DAM (DAM1 and DAM2 clusters)
Ellwanger et al., 2021	5xFAD	scRNA-seq	Cortex	DAM, IFN-R, MHC and cycling-M
Safaiyan et al., 2021	5xFAD	scRNA-seq	Frontal cortex, corpus callosum, optical tracts and medial lemniscus	WAM (parts of DAM gene signature)

*Blue, downregulated signature genes; red, upregulated signature genes. DAM, disease-associated microglia; ARM, activated response microglia; IRM, interferon response microglia; WAM, white matter associated microglia; IFN-R, interferon responsive cluster; MHC, MHC expressing cluster; Cycling-M, (G)2/M phase enriched cluster (proliferating microglia).*

**TABLE 2:** Microglia phenotypes in patients with Alzheimer’s disease revealed by single-cell technologies.

References	Patients	Method	Brain region	Microglia state/cluster (gene signature)
Del-Aguila et al., 2019	3 patients with Mendelian or sporadic AD	snRNA-seq	parietal lobes	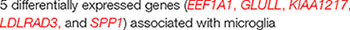
Mathys et al., 2019	48 AD patients	sRNA-seq	Prefrontal cortex	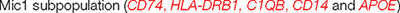
Grubman et al., 2019	6 AD patients	sRNA-seq	Entorhinal cortex	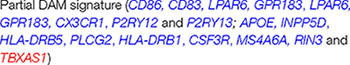
Zhou et al., 2020	11 AD patients with *TREM2*-*CV* and 10 bearing *TREM2-R62H*	snRNA-seq	Dorsolateral prefrontal cortex	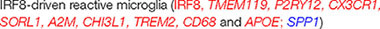
Lau et al., 2020	12 AD patients	snRNA-seq	Prefrontal cortex	Reduced microglia subpopulation that expresses genes which participate in synaptic pruning (*C1QA*, *C1QB* and *C1QC*, encoding complement component 1q) or encode cytokine receptors (*IL4R* and IL1RAP)
Nguyen et al., 2020	15 AD patients	snRNA-seq	Dorsolateral prefrontal cortex	4 microglia subpopulations: homeostatic, motile, amyloid responsive, and dystrophic microglia
Gerrits et al., 2021	10 AD donors with only Aβ pathology or both Aβ and tau pathology	snRNA-seq	Occipital or occipitotemporal cortex	AD1 microglia population express DAM genes, and are enriched with AD risk genes and correlated with tissue Aβ load; AD2 microglia express glutamate receptor *GRID2* and are correlated with tau pathology

*Blue, downregulated signature genes; red, upregulated signature genes. AD, Alzheimer’s disease; DAM, disease-associated microglia; Aβ, β-amyloid.*

## Microglia in Alzheimer’s Disease at Single-Cell Resolution

Due to the high plasticity of microglia, their homogeneity at homeostasis can be readily disrupted under pathological conditions. Through rapid change in gene expression, microglia react in response to surrounding perturbations. It has been challenging to define the cellular heterogeneity of microglia in AD onset and progression (Hickman et al., 2018; Song and Colonna, 2018; Prinz et al., 2019; Bartels et al., 2020; Leng and Edison, 2021). The advancement of single-cell technologies has facilitated the study of microglia biology by uncovering heterogeneous cell states and their underlying molecular pathways within CNS. Microglia states can now be defined by the expression profiling of specific gene sets that are differentially expressed and used to describe cell subpopulations. Single-cell transcriptomic technologies enable unbiased characterization of microglia subtypes and states during transition from normal to disease and response to therapies (Gerrits et al., 2020; Masuda et al., 2020; Boche and Gordon, 2021; Chen and Colonna, 2021). The comprehensive genome-wide analysis by scRNA-seq and other single-cell technologies helps to systematically resolve microglia heterogeneity in AD. Single-cell analysis can also further identify signaling pathways, regulatory factors and potential markers related to microglia, thus providing more insights into microglial response in AD (Olah et al., 2020; Boche and Gordon, 2021; Chen and Colonna, 2021; Provenzano et al., 2021; Summarized in Tables 1, 2).

### Deep Phenotyping of Microglia in Alzheimer’s Disease Mouse Models

**FIGURE 1:**
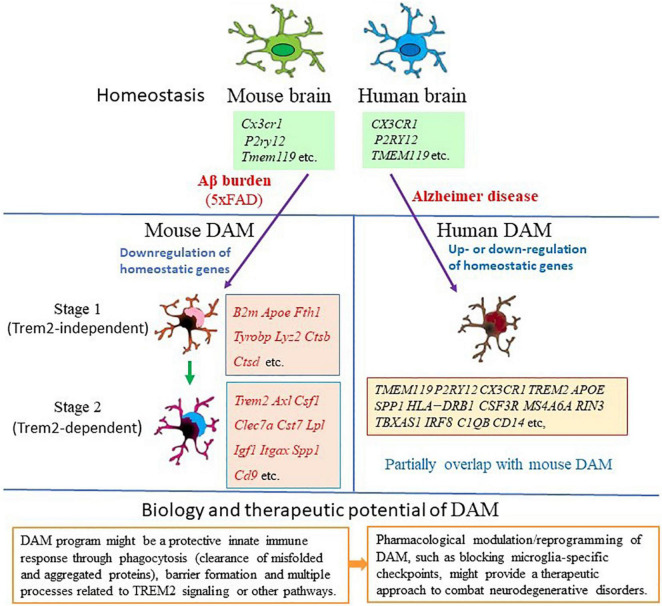
Gene expression features and biological function of disease-associated microglia (DAM). Mouse DAM were first identified in the brains of 5xFAD mouse model for Alzheimer’s disease (AD). Microglia switch from homeostatic to stage 1 DAM (Trem2-independent) and stage 2 DAM (Trem2-dependent) following signals associated with AD pathology, such as Aβ accumulation (Keren-Shaul et al., 2017). DAM are Alzheimer’s disease-associated phagocytic cells conserved in mice and human. In human AD brain, DAM shares gene expression features with mouse DAM (Grubman et al., 2019; Mathys et al., 2019; Zhou et al., 2020; Gerrits et al., 2021). This unique type of microglia has the potential to restrict neurodegeneration, thus may have implications for therapeutics of AD and other neurodegenerative diseases. Key genes involved in each condition are listed in the relative box. Red color indicates upregulation of the gene in the specific stage.

A seminal transcriptomic study in AD mouse models has shown that the disease progression is paralleled in microglia by a gradual transition from a homeostatic state to a disease-associated state, thus shed new light onto the dynamic regulation of microglia in AD brain (Keren-Shaul et al., 2017; Table 1). Using massively parallel scRNA-seq (MARS-seq), Keren-Shaul et al. (2017) mapped the immune cells (CD45^+^) in mouse brains and identified microglia clusters with distinct gene expression profiles, that were referred to as neurodegenerative disease-associated microglia (DAM), in the cortical regions of 5XFAD (AD transgenic mouse model that expresses five human familial AD gene mutations) mouse brains where Aβ plaques are massively deposited. Compared to homeostatic microglia, DAM demonstrate a reduction in the expression of microglia homeostatic genes, such as *P2ry12/P2ry13, Cx3cr1, Cst3*, *Cd33*, *Csf1r*, and *Tmem119*, and upregulation of a vast array of genes, including multiple known AD risk genes (*Apoe*, *Lpl*, *Trem2*, *Tyrobp*, and *Ctsd*) (Keren-Shaul et al., 2017; Table 1 and Figure 1). Gene set enrichment analysis of DAM specific genes further revealed their involvement in lysosomal/phagocytic pathways, endocytosis, and regulation of the immune response. Immunohistochemical analysis showed the localization of these microglia subtypes next to Aβ plaques and DAM with intracellular phagocytic Aβ particles in both mouse and human brain slices (Keren-Shaul et al., 2017). These findings corroborate that DAM may directly affect disease progression. In addition, single-cell analysis confirmed the presence of DAM in the spinal cords of a mouse model of amyotrophic lateral sclerosis (ALS), suggesting that DAM represent a general response to neurodegenerative diseases (Keren-Shaul et al., 2017).

Furthermore, analysis of *Trem2*^–/–^ × 5XFAD mice demonstrated that conversion of homeostatic microglia into DAM is a progressive change that occurs through two sequential but distinct stages, the TREM2 independent stage (DAM1) that involves activation of *Tyrobp*, *Apoe*, and *B2m*, and downregulation of microglia checkpoint genes (such as *Cx3cr1* and *P2ry12*/*P2ry1*3), followed by the TREM2 dependent stage (DAM2) involving upregulation of phagocytic and lipid metabolism genes (such as *Cst7*, *Lpl*, and *CD9*) (Keren-Shaul et al., 2017; Figure 1). This indicates the diverse microglia states during AD progression and the complex mechanisms underlying microglia diversity. It is possible that the function of those genes expressed by DAM is needed to mitigate the disease through phagocytosis.

A snRNA-seq analysis of nuclei pooled from the cortex and hippocampus has further demonstrated the presence of Aβ and TREM2 dependent DAM signature in 5XFAD mice (Zhou et al., 2020; Table 1). The dependency of DAM activation on TREM2 has also been confirmed by a recent scRNA-seq study as *Trem2* deletion greatly attenuate the degree of DAM activation in the *TauPS2APP* AD mouse model with both Aβ and tau pathologies (Lee et al., 2021; Table 1). Similarly, an activated state of microglia with transcriptional features of DAM, termed neurodegenerative microglia (MGnD) which is driven by the TREM2-APOE pathway, has been identified in animal models of ALS and multiple sclerosis by bulk RNA-seq of microglia sorted from whole brain (Krasemann et al., 2017). DAM have been found in normal aging and many neurodegenerative disease models, further supporting that this phenotype is not specific for AD (Deczkowska et al., 2018; Brioschi et al., 2019). As DAM have the potential to restrict neurodegeneration by enhancing clearance of misfolded and aggregated proteins, it may have implications for treatment of AD and other neurodegenerative diseases.

Since regulation of mRNA and the encoded protein can differ dramatically and scRNA-seq may detect only abundant transcripts depending on the depth of analysis, microglia need to be characterized at proteomic level to have a better view of the immune landscape within the brain. Taking advantage of high-dimensional proteome analysis using single-cell mass and fluorescence cytometry (CyTOF), in parallel with genetic fate mapping system, Mrdjen et al. (2018) identified a specific subset of reactive microglia associated with aging and AD by extensive surface protein phenotyping (Table 1). Similar to DAM, this subset of microglia are located around Aβ plaques in *APP/PS1* mice; the expression of homeostatic checkpoint markers (Cx3cr1, MerTK, and Siglec-H) are decreased, accompanied by a light increase of major histocompatibility complex (MHC) class-II (MHC-II) expression (Mrdjen et al., 2018). Notably, in addition to increased phagocytosis-associated markers CD11c and CD14, the activation markers (CD86 and CD44) and an inhibitory ligand named programmed death ligand 1 (PDL1) are upregulated in this microglia subset (Mrdjen et al., 2018; Table 1). The phenotypic changes within this specific subset of microglia display a switch from a homeostatic microglial program to a reactive signature with activated and phagocytic profile. Those surface protein markers provided by this study, can be targeted with commercially available antibodies, thus enabling cell isolation for further investigation into roles of microglia in AD.

Interestingly, DAM are not always identical across distinct disease conditions. The conversion from the homeostatic to the activated phenotype of microglia appears to be a continual process, with transition or intermediate populations or subtypes of DAM have been described. Rangaraju et al. (2018) applied weighted co-expression network analysis (WGCNA) to analyze microglial gene expression data (including scRNA-seq data) and revealed distinct molecular heterogeneity (pro-inflammatory and anti-inflammatory phenotypes) within DAM. Pro-inflammatory DAM emerge earlier in mouse models of AD and are characterized by pro-inflammatory genes (*Tlr2*, *Ptgs2*, *Il12b*, and *Il1b*), surface marker *CD44*, potassium channel *Kv1.3* and regulators (*NFkb*, *Stat1*, and *RelA*), while anti-inflammatory DAM express phagocytic genes (*Igf1*, *Apoe*, and *Myo1e*) and surface marker *CXCR*4 with distinct regulators (*LXR*α/β, *Atf*1), and are prominent at later disease stages (Rangaraju et al., 2018). Notably, this study identified specific drug targets for immunomodulation as LXRα/β agonism and Kv1.3 blockade were found to promote anti-inflammatory DAM, inhibit pro-inflammatory DAM and enhance Aβ clearance in AD models (Rangaraju et al., 2018). Thus, understanding of heterogeneity within DAM could provide novel biological insights into microglia diversity and potentially facilitate discovery of immunomodulatory therapeutic targets and drugs for AD.

In addition to DAM, other different subsets of microglia phenotypes exist during AD progression. A scRNA-seq study of individual microglia cells from the hippocampus of CK-p25 mouse model of severe neurodegeneration with AD-like phenotypes during progression of neurodegeneration identified two distinct reactive microglia phenotypes that express type I interferon (IFN I) and IFN II response genes, respectively, while 202 of the 278 genes upregulated in DAM were also found upregulated in late response microglia (Mathys et al., 2017; Table 1). Additionally, the study discovered previously unknown heterogeneity of microglia in their response to neurodegeneration and disease stage specific microglia states, thus revealing the trajectory of cellular reprogramming of microglia in responding to neurodegeneration and the underlying transcriptional programs during AD progression (Mathys et al., 2017). Friedman et al. (2018) used co-regulated gene modules derived from network analysis of bulk transcriptomes of CNS myeloid cells of diverse mouse models (including tauopathy model datasets) to reanalyze microglial scRNA-seq data from AD mouse model. While confirming the presence of DAM in 5xFAD brains, they identified novel microglia subsets, which are distinct from DAM and express IFN-related or proliferation modules, and a module consisting of the immediate early genes *Fos* and *Egr1* (Friedman et al., 2018). This indicates the value of integrating deep bulk transcriptomic findings with single-cell data to further dissect the cellular heterogeneity in microglia. The proliferating microglia were also identified in recent scRNA-seq studies that found a microglia population enriched for cells in growth (G)2/mitotic (M) phase with a proliferation module (Cycling-M cluster) featured by the expression of proliferation markers (Wang et al., 2020; Ellwanger et al., 2021). Further trajectory analysis to address the relationships among all microglia populations showed that homeostatic microglia differentiate through a continuum of progressively activated states, which ultimately branch into four separate trajectories: DAM, IFN-responsive (IFN-R), MHC (both MHC-II and MHC-I genes) expressing, and proliferating (cycling-M) microglia (Ellwanger et al., 2021; Table 1). It will be necessary to verify whether any of these trajectories convert into another at some point. The representation of all four terminal fates was reduced in *TREM2*^R47H^ × 5xFAD mice, indicating a general requirement of TREM2 for microglia activation (Wang et al., 2020; Ellwanger et al., 2021).

It is still an open question whether there is brain region, disease stage or sex specific difference in the microglial responses to Aβ or Tau pathology, and what are the roles of AD risk genes expressed in microglia in those responses. By gene expression profiling of individual microglial cells isolated from cortex and hippocampus of *App* knockin (*App^NL–G–F^*) mouse model over time, Sala Frigerio et al. (2019) identified two main activated microglia states, the activated response microglia (ARM) and IFN response microglia (IRM), that respond to Aβ accumulation and are also present during normal aging (Table 1). ARM are a heterogeneous cluster overexpressing MHC-II (*H2-Ab1* and *Cd74*) and putative tissue repair genes (*Dkk2*, *Gpnmb*, and *Spp1*). They are highly enriched with AD risk genes (*Apoe*, *Ctsb*, *Ctsd*, *Trem2*, *Tyrobp*, and *H2-Eb1*) and develop faster in female mice. Similar activated states were also found in a second AD (*APP/PS1*) mouse model and in human brains. *Apoe*, the major genetic risk factor for AD, is required to regulate those ARM, but not the IRM (Sala Frigerio et al., 2019). The authors concluded that the ARM response is the converging point for aging, sex, and genetic AD risk factors (Sala Frigerio et al., 2019). In amyloid (*APP^swe^*/*PS-1^L166P^*) and tau (Thy-TAU22) transgenic mouse models, single microglia sequencing confirmed that Aβ, not Tau pathology induces marked transcriptional changes in microglia, including increased proportions of ARM with genetic signature enriched for AD risk genes (Sierksma et al., 2020; Table 1). Thus, it appears that microglia respond to amyloid with a consistent signature of gene expression changes, at least in AD mice. These studies demonstrate the plasticity of microglia in responding to different stressors and highlight the importance of defining disease and stage specific microglial responses, which is essential for designing therapeutics to target microglial behaviors in AD in a beneficial way.

It is largely unknown about the specific microglial responses during aging that results in gray and white matter degeneration in the brain. How and to what extent DAM are also generated during normal aging need to be further investigated. As aging-induced damage to the brain involves degeneration of myelinated nerve fibers, characterized by release of lipid-rich myelin debris, it is possible that microglial responses could differ between aged gray and white matter. In characterizing the microglial responses by scRNA-seq analysis of white and gray matter separately, Safaiyan et al. (2021) identified white matter-associated microglia (WAM) as a novel microglia state associated with white matter aging (Table 1). WAM share some of the DAM gene signature and are characterized by downregulation of homeostatic genes, such as purinergic receptor (*P2ry12* and *P2ry13*) and checkpoint genes (*Csfr1r*, *Cx3cr1*, *Hexb*, and *Tmem119*) and by upregulation of DAM associated genes, such as lipid metabolism and phagosome related genes (*ApoE*, *Cst7*, *Bm2*, *Lyz2*, *Cd63*, and *Clec7a*), cathepsins (*Ctsb*, *Ctss*, and *Ctsz*), and MHC-II related genes (*H2-D1* and *H2-K1*) (Safaiyan et al., 2021). WAM gene signature was also observed in re-analysis of existing datasets from previous scRNA-seq studies which analyzed microglia during normal brain aging without reporting WAM (Hammond et al., 2019; Sala Frigerio et al., 2019). WAM are TREM2 and aging dependent, and co-exist with DAM in AD mouse models. Similar to DAM, WAM are generated prematurely, depending on APOE in AD mouse models, while they form independent of APOE in aged brain. Functionally, WAM are engaged in clearing degenerated myelin (Safaiyan et al., 2021). As WAM may represent a protective response required to clear myelin debris that accumulate during aging and disease, enhancing formation of WAMs could have therapeutic value to help to combat the aging and AD. Future studies will need to confirm that that WAM also exist in humans.

### Microglia Phenotypes in Alzheimer’s Disease Patients

Single-cell transcriptomic analysis of brain samples from AD patients indicated that all major brain cell types could be affected by AD pathology (Mathys et al., 2019; Zhou et al., 2020; Gerrits et al., 2021). As studies have shown concordance between single-cell and single-nucleus transcriptome profiles, snRNA-seq is becoming a tool for studying cellular transcriptional heterogeneity in brain tissues particularly for human brain, for which often only frozen material is available. Microglial signatures in human AD brain samples obtained through snRNA-seq show considerable heterogeneity and can differ from the DAM expression signature detected in AD mouse models (Mathys et al., 2019; Alsema et al., 2020; Boche and Gordon, 2021; Chen and Colonna, 2021; Gerrits et al., 2021; Figure 1). An initial snRNA-seq analysis of the brain tissues from three patients with Mendelian or sporadic AD showed that it is possible to identify different cell types from frozen brains of patients with different forms of AD and discovered five differentially expressed genes (*EEF1A1*, *GLULL*, *KIAA1217*, *LDLRAD3*, and *SPP1*) that are consistently associated with microglia in all three samples (Del-Aguila et al., 2019; Table 2). Of these genes, *SPP1* (also referred as osteopontin) is one of the top DAM markers identified in AD mouse models and previously used as a marker for immunohistochemical staining of microglia in human brain (Keren-Shaul et al., 2017; Del-Aguila et al., 2019). As the results are encouraging, it is possible to better characterize the expression profile and trajectories of microglial cells in AD patients by increasing the number of samples to sequence enough microglial cells. Mathys et al. (2019) analyzed 80,660 single-nucleus transcriptomes from the prefrontal cortex of 48 individuals with AD and identified the AD pathology-associated Mic1 cell subpopulation in which the marker genes, including the MHC-II genes (*CD74* and *HLA-DRB1*), significantly overlapped with those of mouse DAM (Table 2 and Figure 1). The presence of a subpopulation of microglia that highly express MHC-II proteins was further confirmed by immunohistochemistry in the brain of AD patients. Interestingly, this microglia subpopulation in humans express AD-associated genes that are not seen in the animal models, including the complement component *C1QB* and the pattern recognition receptor *CD14.* Many of Mic1 marker genes, such as *APOE*, are specific to AD pathology, but not identified in aged microglia (Mathys et al., 2019; Table 2). Thus, the Mic1 subpopulation appears to be AD specific and represents a distinct microglia state that shares features with, but differs from microglial cell states in mouse AD models (Mathys et al., 2019).

The discrepancies between mouse and human data were also observed in other studies. In surveying gene expression changes in human AD by snRNA-seq, Zhou et al. (2020) identified a microglia signature that is reminiscent of IRF8-driven reactive microglia in peripheral nerve injury (Table 2). In this microglia cluster, the homeostatic genes (*TMEM119*, *P2RY12*, and *CX3CR1*) are actually upregulated in AD, along with increased expression of the transcription factor IRF8. Other genes previously known to be upregulated in human AD, but not as part of the mouse DAM signature, including *SORL1, A2M*, and *CHI3L1*, are also highly upregulated. MHC-II related genes, *TREM2, CD68*, and *APOE* are among the few DAM gene homologs upregulated in human AD samples. Other DAM genes were not detected (*CST7*, *GPNMB*, and *LPL*) or found either unchanged (*TYROBP*) or even downregulated (*SPP1*) in human AD microglia in this study (Zhou et al., 2020; Table 2 and Figure 1). These data suggest that the signature of human microglia in AD is distinct from that of DAM in AD mouse models (Figure 1). Notably, the reactive phenotype of microglia was observed less evident in *TREM2* mutant carriers than in non-carriers, demonstrating that TREM2 is required in both mouse and human AD, despite the species specific differences (Zhou et al., 2020).

Subsequent transcriptomic analyses of human AD also reported an incomplete DAM signature. Grubman et al. (2019) applied snRNA-seq to analyze entorhinal cortex samples of AD patients and found that AD microglia downregulate genes related to cell–cell adhesion (*CD86* and *CD83*), lipid response (*LPAR6*), G-protein-coupled receptor pathways (*GPR183* and *LPAR6*), and homeostatic genes, such as *CX3CR1*, *P2RY12*, and *P2RY13*, while the AD risk gene *APOE* is upregulated as has been previously described in AD mouse models (Table 2 and Figure 1). Several previously described microglia specific AD risk genes, including *INPP5D*, *HLA-DRB5*, *PLCG2*, *HLA-DRB1*, *CSF3R*, and *MS4A6A*, are highly specifically expressed in microglia. Additionally, two microglia specific AD risk genes not previously associated with microglia, *RIN3* and *TBXAS1*, were detected in this study (Grubman et al., 2019; Table 2). The study supports further detailed functional investigation in a microglial model to better understand the contribution of these genes to AD. In single-nucleus transcriptomic analysis of the prefrontal cortical samples of AD patients, Lau et al. (2020) found that the AD samples exhibited a reduced proportion of microglia subpopulation that expresses genes which participate in synaptic pruning (*C1QA*, *C1QB*, and *C1QC*, which encode complement component 1q) or encode cytokine receptors (*IL4R* and IL1RAP) (Table 2). The results suggest that the loss of this typical microglia subpopulation might contribute to the imbalanced complement signaling and synaptic pruning in AD. Using an unbiased snRNA-seq approach and a novel bioinformatics pipeline to characterize postmortem human AD brains, Nguyen et al. (2020) identified four key microglia subpopulations: homeostatic, motile, amyloid responsive, and dystrophic microglia (Table 2). Among them, the homeostatic subpopulation demonstrates the upregulation of *CX3CR1*, while other homeostatic marker genes, such as *TMEM119* and *P2RY12*, are not changed, likely due to the sparsity of data inherent to snRNA-seq. Potential marker genes were also identified for the other three microglia subpopulations, such as *FGD4*, *FTL*, and *CD163* for motile, dystrophic, and amyloid responsive microglia, respectively (Nguyen et al., 2020; Table 2). Amyloid responsive microglia specifically express CD163, a transmembrane scavenger receptor that is part of the scavenger receptor cysteine-rich (SRCR) domain family and has a variety of immunoregulatory functions. Amyloid responsive microglia are conceptually similar to reactive mouse microglia populations (DAM, MGnD, or ARM) in their physical association with Aβ plaques, suggesting that amyloid responsive microglia may act as a defense against Aβ accumulation. While most genes do not overlap between mouse DAM and human amyloid responsive microglia, human amyloid responsive microglia and activated mouse microglia do share some similarities including the increased expression of APOE, accompanied by the decrease of TREM2 expression in amyloid responsive microglia. Notably, CD163 positive amyloid responsive microglia are depleted in patients with *APOE* and *TREM2* mutant variants, supporting that these genetic risk factors may confer risk for AD by down-regulation of the amyloid responsive microglia response (Nguyen et al., 2020). These studies imply that while discrepancies exist, at least some of the shared DAM genes could reflect conserved patterns of microglial responses to AD pathology between human and mouse microglia signatures.

It is still largely unknown how Aβ and tau pathology could affect human microglia transcriptional profiles. A recent study performed snRNA-seq on 482,472 nuclei from non-demented control brains and AD brain regions containing only Aβ plaques or both Aβ plaques and tau pathology (Gerrits et al., 2021). While homeostatic microglia expressing P2RY12 and CX3CR1 were found, the study identified two distinct AD pathology-associated microglia populations. Of them, the phagocytic/activated AD1 microglia population express DAM genes, are localized to Aβ plaques, and their abundance are correlated with tissue Aβ load; the AD2 microglia express the gene for glutamate receptor GRID2, are more abundant in samples with tau pathology, and their presence are correlated with tissue phosphorylated tau load (Gerrits et al., 2021; Table 2 and Figure 1). Interestingly, *CD163* is expressed and exclusively enriched in AD1 microglia. Of the 63 AD risk genes expressed in microglia, 15 are significantly enriched and highly expressed in AD1 microglia, and only six genes are moderately enriched in AD2 microglia (Gerrits et al., 2021). This finding is consistent with a recent mouse study that has shown that the genetic risk of AD is functionally associated with the microglia response to Aβ pathology, not to tau pathology (Sierksma et al., 2020), suggesting that Aβ pathology is upstream of tau pathology, and the immune response of AD1 microglia to Aβ pathology is involved in the onset and progression of AD. This detailed characterization of human AD pathology-associated microglia phenotypes provides new insights into the pathophysiological roles of microglia and potentially offers new microglia state specific therapeutic targets for AD.

The discrepancies between human microglial signatures and their mouse counterparts could be explained by the fact that human brain samples usually represent a terminal stage of AD with amyloid and tau pathology, as well as extensive neuronal loss, while mouse models might just recapitulate either earlier stages of the disease characterized by Aβ accumulation or frontotemporal dementia–like tauopathy without amyloidosis (Alsema et al., 2020; Masuda et al., 2020; Boche and Gordon, 2021; Chen and Colonna, 2021; Provenzano et al., 2021). There are many other biological reasons, such as the different brain regions from where the samples are taken, and the genetic background or ethnic origins of AD patients cohorts analyzed, that could contribute to variations in human AD profiles from different studies. Technically, almost all microglial profiles in human AD were determined by snRNA-seq, while mouse DAM were mainly identified by scRNA-seq. The number of microglia nuclei sequenced may be insufficient to identify a DAM cluster in humans. This may partially explain human–mouse discrepancies in the AD transcriptomic profiles. Additionally, the overall quality of human RNA samples could be poorer than mouse samples due to postmortem intervals preceding human sample collection and processing. Further improvement in tissue collection and processing would potentially reduce variations in human samples and human–mouse discrepancies (Alsema et al., 2020; Lewcock et al., 2020; Masuda et al., 2020; Boche and Gordon, 2021; Chen and Colonna, 2021; Gerrits et al., 2021; Provenzano et al., 2021). Notably, a comparison of nuclear (snRNA-seq) and total cellular transcriptomes (scRNA-seq) of human microglia in a study revealed that a small population of genes is depleted in nuclei, while most genes show similar relative abundances in cells and nuclei (Thrupp et al., 2020). This small population is enriched for genes known to be involved in microglial activation, such as *APOE*, *CST3*, *FTL*, *SPP1*, *B2M*, *PLD3*, and *CD74*, containing 18% of previously identified microglial disease-associated genes (Thrupp et al., 2020). The low sensitivity of snRNA-seq to detect activation genes is likely responsible for the difficulty in identifying a consistent activation signature in the human brain in snRNA-seq based studies.

## Challenges and Emerging Approaches for Studying Microglia in Alzheimer’s Disease

The discrepancies between human AD and mouse models demonstrated by sc/snRNA-seq studies of microglia states, highlights the need to study microglia biology in human cells. Multiple approaches have been developed to study human microglia at AD conditions and understand the impact of AD risk genes on the functions of microglia. One approach is the development of human *in vitro* disease models using microglia or brain organoid generated from human induced pluripotent stem cells (iPSCs) and embryonic stem cells (ESCs) in combination with molecular genetic techniques (such like CRISPR) to enable deletion, mutation, or overexpression of disease genes (Wang, 2018; Masuda et al., 2020; Chen and Colonna, 2021; Provenzano et al., 2021). AD like gene expression signatures have been observed in microglia derived from human ESCs harboring AD mutant *SORL1* and *TREM2* introduced by CRISPR-Cas9 editing (Liu et al., 2020). The regulatory function of TREM2 could be confirmed by comparing microglia differentiated from wild-type and isogenic *TREM2* knockout human iPSCs, suggesting that these cells can be applied to study AD related disease settings (Reich et al., 2021). To overcome the challenge faced by *in vitro* cell culture systems to recapitulate key aspects of the complex CNS microenvironment, microglia derived from human iPSCs or ESCs have been transplanted into mouse brain (Hasselmann et al., 2019; McQuade et al., 2020; Fattorelli et al., 2021). While retaining a transcriptome profile distinct from that of endogenous mouse microglia, transplanted microglia can exhibit transcriptional responses to Aβ plaques that only partially overlap with that of mouse microglia, revealing human specific Aβ responsive genes (Hasselmann et al., 2019; Fattorelli et al., 2021). Moreover, single-cell sequencing of transplanted human microglia has revealed a loss of DAM responses in human *TREM2* knockout microglia, highlighting TREM2 dependent DAM signatures (McQuade et al., 2020). These models will provide new opportunities to study human microglia diversity in AD mouse models and also present an opportunity to test specific therapeutic for AD at more humanized conditions.

While genetic studies have identified many AD risk gene variants that potentially affect microglia, understanding the regulatory relationship between transcription factors and their target genes is key to unveiling gene expression programs in microglia that regulate disease progression. The genome-wide analyses of chromatin accessible regions and histone modifications combined with single-cell analyses will help us to dissect genetic regulatory mechanisms underlying microglia diversity and understand how microglia is affected by AD risk variants (Gosselin et al., 2017; Colonna and Brioschi, 2020; Masuda et al., 2020; Young et al., 2021). The detailed information on gene regulation in human and mouse microglia might also help to explain at least some of the discrepancies between human AD and mouse models.

Finally, one limitation of scRNA-seq and snRNA-seq analyses is that they cannot define the precise location of microglia subsets and signatures within CNS niches and potential interactions with other cells, as spatial context is lost due to cell or nuclei isolation. Fortunately, recent development of spatial single-cell omics technologies has overcome this limitation and allowed the simultaneous collection of gene expression and spatial information (Masuda et al., 2020; Chen and Colonna, 2021; Provenzano et al., 2021). For instance, spatial transcriptomics has been applied to study *App^NL–G–F^* mice, which confirmed the association between DAM and amyloid plaques (Chen et al., 2020). Strategies that combine multiplexed fluorescence *in situ* hybridization with sequencing have also been developed by imprinting RNAs with oligo-conjugated barcodes that are measured through sequential rounds of hybridization and super-resolution imaging (Eng et al., 2019; Kim et al., 2019; Su et al., 2020; Boche and Gordon, 2021; Provenzano et al., 2021). These technologies have made it possible to identify new cell clusters/populations while maintaining their spatial organization and information about subcellular mRNA localization patterns as well as intercellular connections. Their application to research in microglia biology will provide information on individual cells within the native microenvironment of surrounding cells and AD pathology (Chen and Colonna, 2021; Provenzano et al., 2021).

## Conclusion

In general, the studies in mouse models have demonstrated fundamental and relatively consistent profiles of microglial activation in response to AD pathologies. In contrast, human microglia are more complex and heterogeneous (Hashemiaghdam and Mroczek, 2020; Masuda et al., 2020; Boche and Gordon, 2021; Chen and Colonna, 2021; Provenzano et al., 2021). With the existing knowledge of different microglia populations, it will be critical to further investigate the biological functions of these cell populations and determine whether they have a beneficial or detrimental impact on AD progression. The development of new technologies will facilitate the translation of single-cell profiling data into an improved functional understanding of microglia populations in the brain. Particularly, a combination of CRISPR/Cas9-based genome editing and single-cell profiling can provide a powerful tool for the high-throughput dissection of gene functions in different microglia subsets. In addition, determination of how each cell population respond to microglia targeting therapies will be able to more comprehensively assess their therapeutic efficacy.

Encouragingly, high-throughput single-cell analysis in neurodegenerative diseases have currently been extended far beyond microglial cells. A wealth of single-cell transcriptome datasets are now available for other glial cells, such as astrocytes and oligodendrocytes, as well as neurons (Jordao et al., 2019; Mathys et al., 2019; Habib et al., 2020; Zhou et al., 2020; Leng et al., 2021). With the advancement of spatial single-cell omics platforms, integration of microglia data with the analyses of other major brain cell types will help to come out a detailed picture of cellular responses to AD pathologies at relevant spatial contexts and potentially open new avenues for the development of therapeutics for AD.

## Author Contributions

HW conceived, wrote, and revised the manuscript.

## Conflict of Interest

The author declares that the research was conducted in the absence of any commercial or financial relationships that could be construed as a potential conflict of interest.

## Publisher’s Note

All claims expressed in this article are solely those of the authors and do not necessarily represent those of their affiliated organizations, or those of the publisher, the editors and the reviewers. Any product that may be evaluated in this article, or claim that may be made by its manufacturer, is not guaranteed or endorsed by the publisher.
